# Growth differentiation factor 5 improves meniscal healing in a pilot study on rats

**DOI:** 10.1002/jeo2.70309

**Published:** 2025-06-12

**Authors:** David Mazy, Nathaniel Léveillé, Line Séguy, Irene Londono, Florina Moldovan, Marie‐Lyne Nault

**Affiliations:** ^1^ CHU Sainte‐Justine Montréal Québec Canada; ^2^ Department of Surgery Université de Montréal Montréal Québec Canada; ^3^ CHU Sainte‐Justine Azrieli Research Center Montréal Québec Canada; ^4^ Faculty of Pharmacy Université de Montréal Montréal Québec Canada; ^5^ Department of Orthopedic Surgery CIUSSS Hôpital du Sacré‐Cœur de Montréal (HSCM) Montréal Québec Canada

**Keywords:** animal study, biologic augmentation, growth differentiation factor 5, growth factor, meniscal healing, meniscal repair

## Abstract

**Purpose:**

Meniscus injuries are common, but failed repairs remain an issue. The aim of this study was to demonstrate, in an in vivo rat model, the ability of growth differentiation factor 5 (GDF5) to improve meniscal tear healing.

**Methods:**

Eight Lewis rats (four females and four males) underwent radial tear of the medial meniscus on the right knee. There were two post‐operative treatments: the GDF5 group (*n* = 4) received 0.1 mg/mL of GDF5, and the saline group (*n* = 4) a saline injection. The eight left knees were the control without surgery. Sacrifice was six weeks post‐operatively. The GDF5 and saline groups were compared according to histology and meniscus healing score (MHS) in different zones: red‐red (R‐R), red‐white (R‐W) and white‐white (W‐W).

**Results:**

In the R‐R zone, the median [interquartile range, IQR] MHS was 2.5 [2–3] in the GDF5 group and 2 [1.25–2] in the saline group (*p* = 0.200), and in the W‐R zone it was 2 [2–2.75] for GDF5 and 1 [1–1.75] for saline (*p* = 0.047). There was no difference in the W‐W zone (median MHS under one; *p* = 0.686). Regardless of groups, median [IQR] MHS in R‐R (2 [2–2.75]) and R‐W zones (2 [1–2]) were significantly higher (*p* < 0.001) than in the W‐W zone (0 [0–1]). MHS intraclass correlation coefficient inter‐observer was 0.88 and intra‐observer was 0.90.

**Conclusions:**

GDF5 increases meniscal healing, especially in the R‐W zone, although the W‐W zone remains challenging. GFD5 is a promising factor for improving meniscus healing. The small sample size and absence of biomechanical evaluation are limitations that warrant caution when interpreting these findings. Further studies with larger sample sizes in larger animal models, combined with meniscal repair, are required to confirm these preliminary results.

**Study Design:**

Animal laboratory study.

**Level of Evidence:**

Level V, animal study.

**Abbreviations**
GDF5: growth differentiation factor 5
MHS: meniscal healing score
OA: osteoarthritis
R‐R: red‐red
R‐W: red‐white
TGF‐beta: transforming growth factor‐beta
W‐W: white‐white

## INTRODUCTION

The meniscus is an essential component of the knee joint, providing shock absorption, load distribution and lubrication, and playing a proprioceptive role [3]. At birth, the meniscus is almost completely vascularized, but as it grows, its vascularization becomes limited to the most peripheral area [11]. Unfortunately, meniscus injury often leads to orthopaedic surgery, with more than 750,000 operations per year in the United States alone [2]. Over the last few decades, it has been clearly demonstrated that meniscal preservation, through surgery and suturing, can prevent early osteoarthritis (OA) caused by meniscectomy [5, 35]. Nevertheless, the rate of secondary meniscectomies can reach up to 24%, essentially due to failed repair [4, 24]. Consequently, there have been various attempts to find a factor that can improve meniscal healing in the form of biological augmentation. The factors most frequently used are platelet‐rich plasma, fibrin clots and mesenchymal stem cells [15, 20]. Results remain inconclusive, and further studies are needed to establish their efficacy and support their routine clinical use [15, 20]. Other factors, such as bone morphogenetic protein‐7, fibroblast growth factor‐2 and angiogenin, have also been investigated, but the results so far have not shown improved meniscus healing [26].

Growth differentiation factor 5 (GDF5), also known as cartilage‐derived morphogenetic protein 1, is a member of the transforming growth factor‐beta (TGF‐beta) superfamily [18]. It is closely related to bone morphogenetic proteins, which are also part of the TGF‐beta family. During embryonic development, GDF‐5 plays an important role in the formation of joints, long bones and tendons [38]. This factor also contributes to the maintenance and repair of bones, cartilage and other soft tissues of the synovial joint [36]. GDF5 has already been studied in other articular tissues, such as cartilage, but there are no studies on its use in meniscal tissue [27, 29, 37]. GDF5 has the distinctive advantage of inducing local cells to differentiate specifically into fibro‐chondrocytes, thus promoting the formation of fibrocartilaginous extracellular matrix, theoretically even in the avascular zone [9, 29]. Investigating GDF5 aims to address a critical gap in the current literature regarding targeted biological approaches for enhancing meniscal healing through the stimulation of local cellular differentiation and extracellular matrix synthesis.

The aim of this study was to investigate the use of GDF5 to promote meniscal healing in an in vivo rat model. The authors hypothesized that intra‐articular injection of GDF5 would result in accelerated healing of the meniscus in vascular and avascular areas.

## MATERIALS AND METHODS

This study follows the ARRIVE 2.0 guidelines for reporting animal research [30].

### Animals

This study was approved by the Animal Care and Experimentation Committee of CHU Sainte‐Justine hospital in Montreal, Canada (project #2024‐5181, 15 June 2023). This committee prepared and approved a study protocol—all procedures adhered to the guidelines of the Canadian Council on Animal Care [34]. Every effort was made to follow the principles of reduction, replacement and refinement. The sample size was determined based on institutional limitations for a pilot study, in accordance with the principle of reduction. Eight‐week‐old male (*n* = 4) and female (*n* = 4) Lewis rats of body weight 150–250 g were purchased from Charles River Canada and housed under standard conditions at a controlled temperature (24°C) light/dark room. Same‐sex animals were housed in the same cage. They were fed a standard commercial diet and had unlimited access to tap water. There was no discrimination in terms of sex, and both male and female specimens were used.

### Surgery

**Figure 1:**
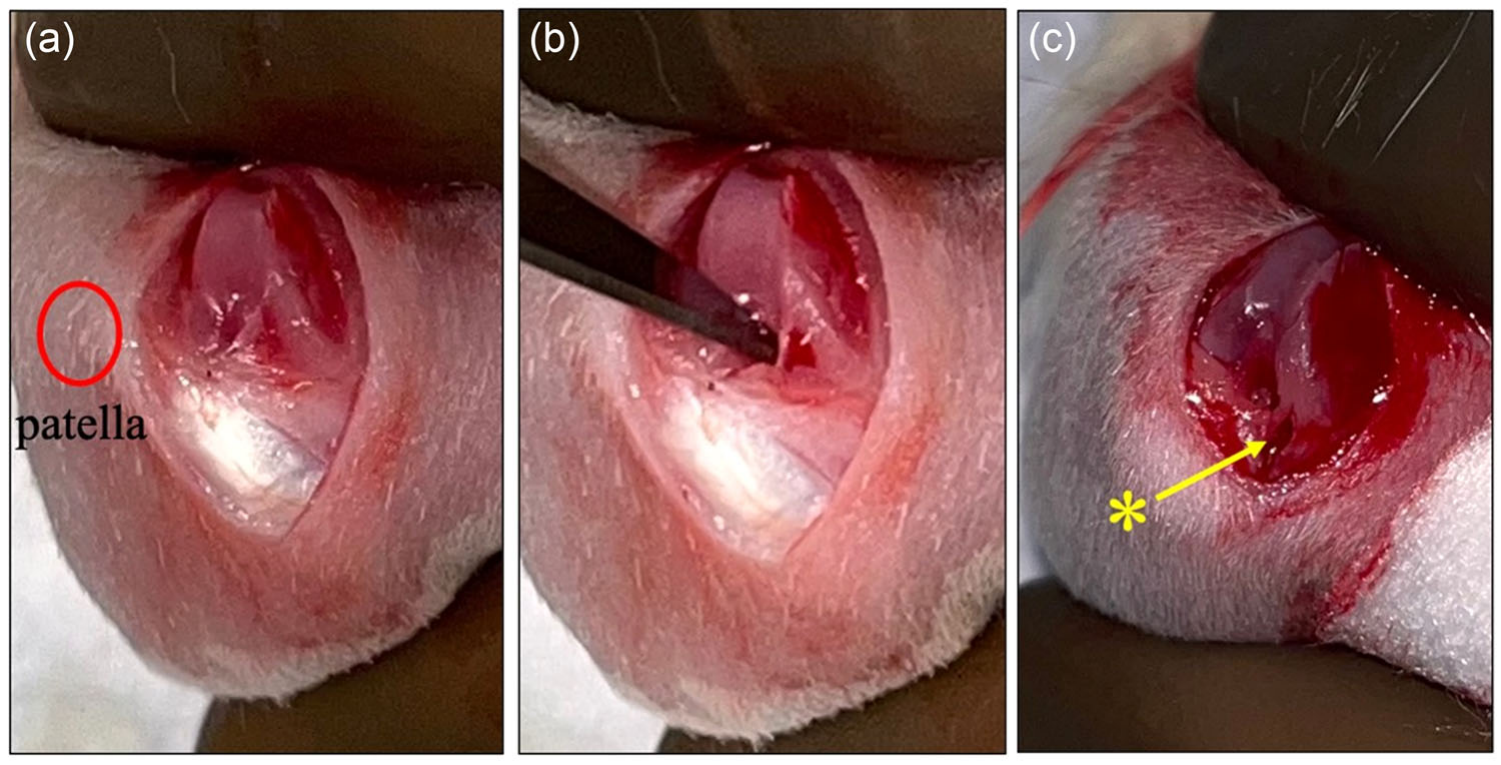
(a) Intra‐articular view of the right knee after patellar lateral dislocation (red circle). (b) Performing an anterior horn radial tear of the internal meniscus. (c) Radial tear (*).

All rats underwent meniscal tear surgery. Preoperative analgesia with buprenorphine (0.05–0.1 mg/kg SC, 25G needle) was performed, followed by mask‐inhaled isoflurane anaesthesia (2%–3%/L O_2_). Only the right back leg was operated on. After shaving the operative site and conventional disinfection, a median longitudinal para‐patellar incision was made. Deep plan incision was followed by lateral patellar dislocation. A complete full‐thickness radial tear was performed with a scalpel blade no. 11 on the anterior horn of the medial meniscus to separate the meniscus into two parts. The procedure was performed with the knee in full flexion (Figure 1). The anterior horn was injured because it was the only possible access to the meniscus without destabilizing the knee completely. The knee was flushed with saline, and sutures were performed in two separate planes. The skin was closed with surgical glue (Glustitch® cyanoacrylate) and staples to seal the closure. The eight Lewis rats were then divided into two groups. One group (GDF5 group) of four rats received a 50‐µL intra‐articular injection of GDF5 (Human GDF‐5 Recombinant Protein) at a concentration of 100 µg/mL, and the other group (saline group) received a 50‐µL saline injection. The concentration chosen in our study resulted in an administered total dose of 5 µg, which is lower than doses previously used in cartilage studies, typically ranging from 30 to 100 µg per injection [29, 37]. Given the lack of available evidence regarding optimal dosing for meniscal healing, we deliberately selected a low dose as an initial exploration, prioritizing safety and aiming to investigate whether even minimal concentrations could yield beneficial effects in meniscal tissue. The injections were performed right after surgery with a 0.5‐mm diameter, 25G subcutaneous needle. Carrying out the injection with the needle placed in the joint before closure enabled us to ensure intra‐articular injection, which is not easy to achieve outside of surgery, given the small size of the joint.

Post‐operative analgesia with buprenorphine (0.05–1 mg/kg subcutaneous) was administered for 48 h as needed. Rats also received post‐operative hydration (saline injection 10 mL/kg subcutaneous) immediately, and as required up to 8 h post‐operative. Surgery and recovery were performed over a heated mattress. Wakefulness, agitation, hydration and respiratory rate were monitored post‐operatively. Rats in both groups were allowed to move and feed freely in the cages. In the event of premature death or an open joint wound, the animal would be excluded from the study. In the event of death, the cause would be investigated.

### Sample collection and preparation

**Figure 2:**
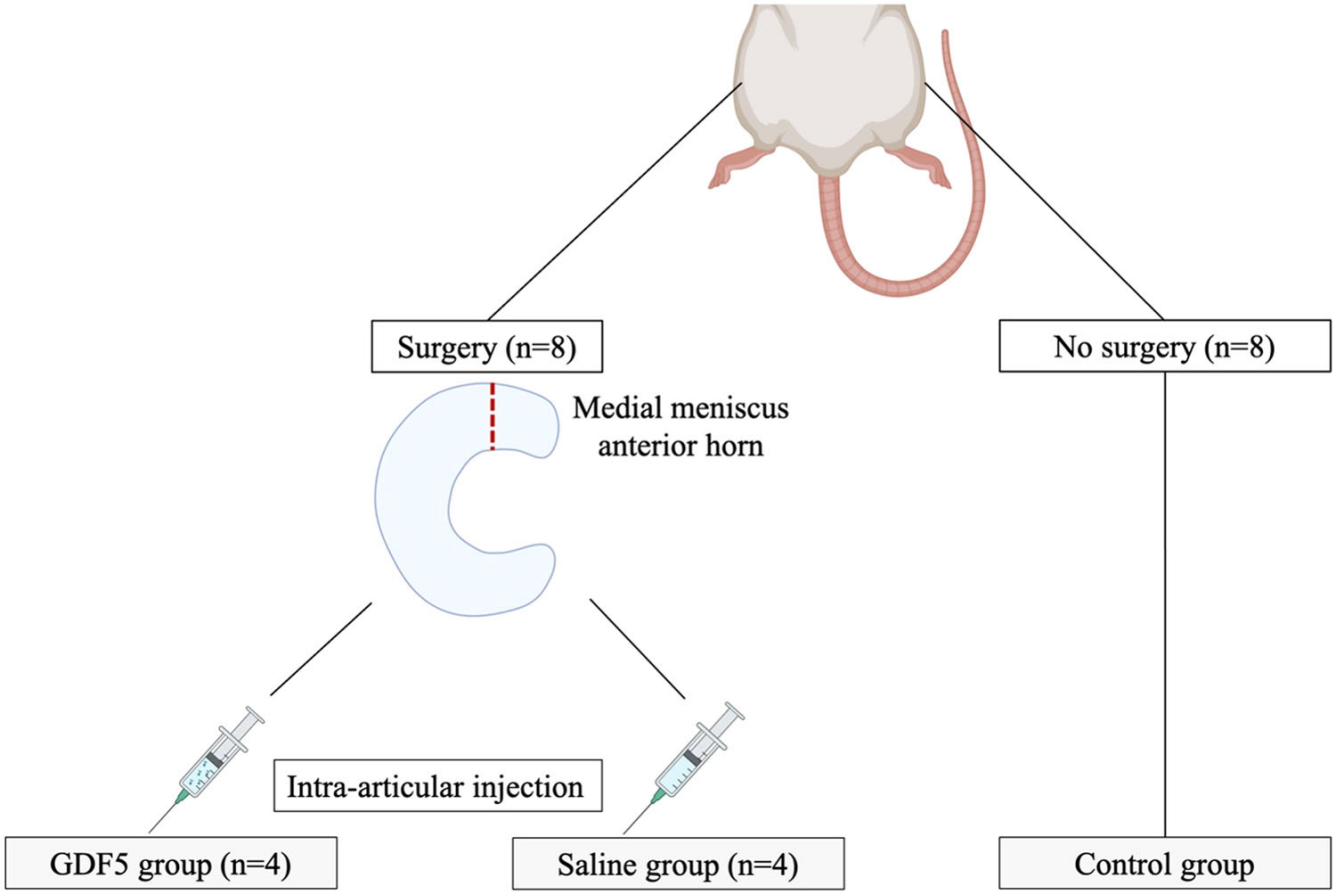
Experimental plan. GDF5, growth differentiation factor 5.

Although there were no studies on the exact timeframe for GDF5 action, several reports have suggested a timeline of 6 weeks before sacrifice [19, 38]. In this study, rats were euthanized at 6 weeks post‐operatively. Next, both knees were harvested and their superficial soft tissue envelop was cleaned. Then, they were immediately immersed in the cross‐linking fixative formalin for 24 h to stabilize the tissue architecture for histological assessment. The specimens were then immersed in a neutral decalcification solution for 5 days at room temperature, circulated, and embedded in paraffin. The eight left knees were used as a control group without surgery to confirm the absence of spontaneous meniscal lesions in the absence of surgery (control group). No additional sham‐operated group was included due to ethical considerations aiming to minimize animal use, consistent with the principle of reduction, and because of practical constraints inherent to this pilot study [12]. Details of the experiment are shown in Figure 2.

### Histological evaluation

**Figure 3:**
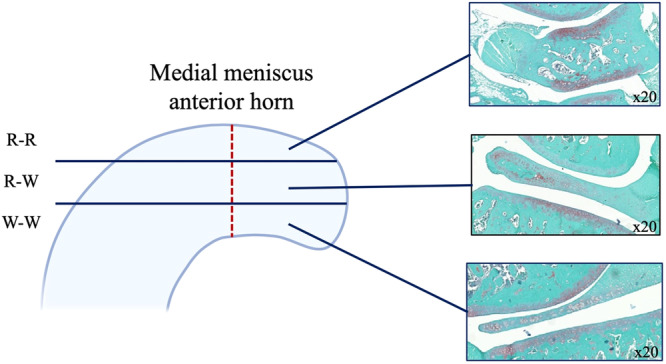
Sections of the radial tear were divided according to the three vascular zones with histological findings of the control group. R‐R, red‐red; R‐W, red‐white; W‐W, white‐white.

We used samples obtained 6 weeks after surgery. The formalin‐fixed paraffin‐embedded tissue samples were sectioned using a heavy‐duty microtome. Five‐micron serial tissue sections perpendicular to the tear were acquired from the knee joint. A 20 μm spacing was maintained between each pair of slides. Histomorphological staining was performed as previously described [32]. Slides were deparaffinized, rehydrated, stained with Safranin O (Sigma‐Aldrich, catalogue #S2255. Which colours proteoglycans red), counterstained with Fast Green FCF (Sigma‐Aldrich, catalogue #F7258‐25G. Which colours proteins green) and with Weigert's hematoxylin (ACP Chemicals Inc. #517‐28‐2. Which colours nuclei black), dehydrated, cleared, and mounted in Permount (Fisher Scientific, catalogue #SP15‐500). Representative digital photomicrographs were acquired with the Axioscan slide scanner. Three different tissue slides for each specimen were selected for analysis. One in the red‐red (R‐R) zone, one in the red‐white (R‐W) zone and the last in the white‐white (W‐W) zone (Figure 3).

The meniscal healing score (MHS) was determined according to the method described by Hashimoto et al. [14]. This score assesses the existence and quantity of extracellular matrix at the site of the meniscal tear. The score ranges from 0 to 3 points. 0 = no significant reaction; 1 = reaction but no bridge connecting the two components; 2 = connective tissue between the components; 3 = explants with fibrous continuity between the two sides of the gap. All samples were independently and blindly evaluated by one orthopaedic surgeon and one biologist (D.M. and N.L.). This was the only stage in which the researchers were blinded to treatment throughout the experiment.

### Statistical analysis

MHS is expressed as median ± interquartile range (IQR) due to small sample sizes and the impossibility of verifying the normality of the distribution of values using the Shapiro–Wilk test. The non‐parametric Mann–Whitney *U* test was used to detect differences between the two groups (saline and GDF5). The intraclass correlation coefficient (ICC) for MHS was evaluated by two different observers. ICC values range from 0 to 1, where a value higher than 0.75 indicates excellent agreement [33]. For the intra‐observer correlation coefficient, observer 1 was blinded on the two separate times the score was completed (with a 4‐week interval). *p* Value < 0.05 indicates statistical significance. The effect size (*r*) was calculated to better quantify the magnitude of the observed effects. All statistical analyses were performed using SPSS (v28.0.1.0; IBM Corp.).

## RESULTS

### Histological evaluation

**Figure 4:**
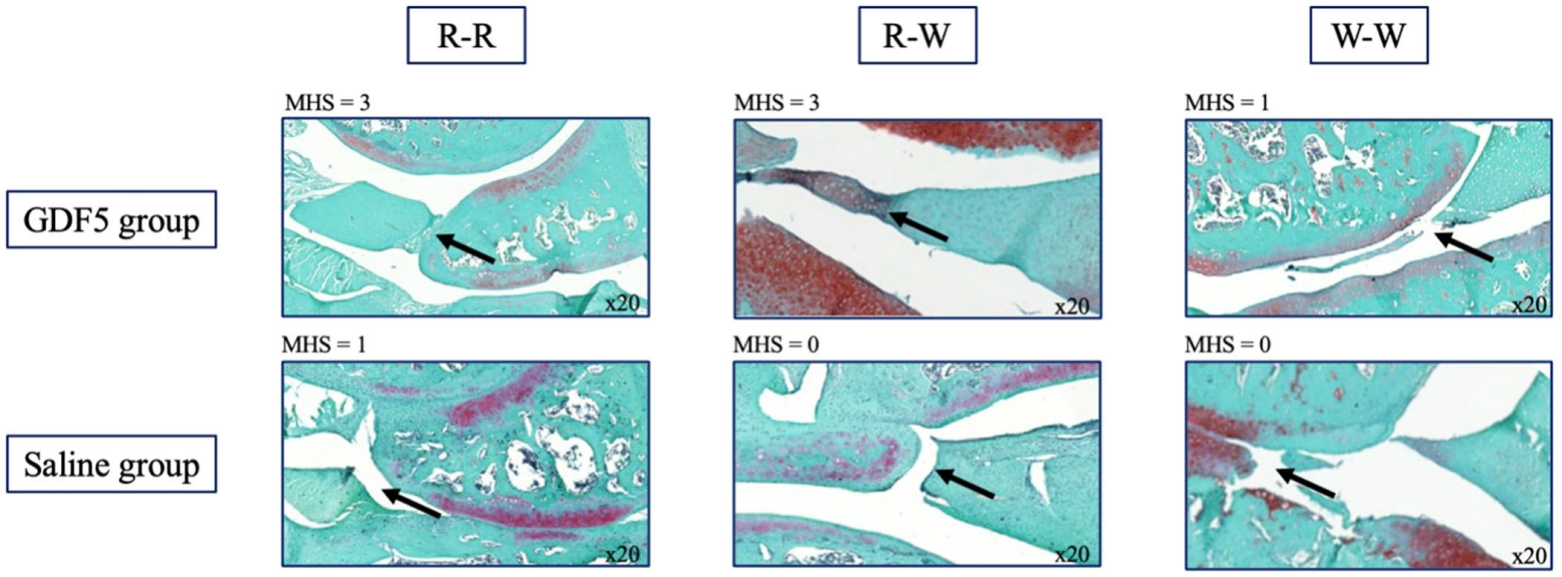
Histological evaluation of meniscal tear and healing. Comparison of GDF5 and saline groups in red‐red (R‐R), red‐white (R‐W) and white‐white (W‐W) zones. Arrows indicate meniscus tears. MHS, meniscal healing score.

**Figure 5:**
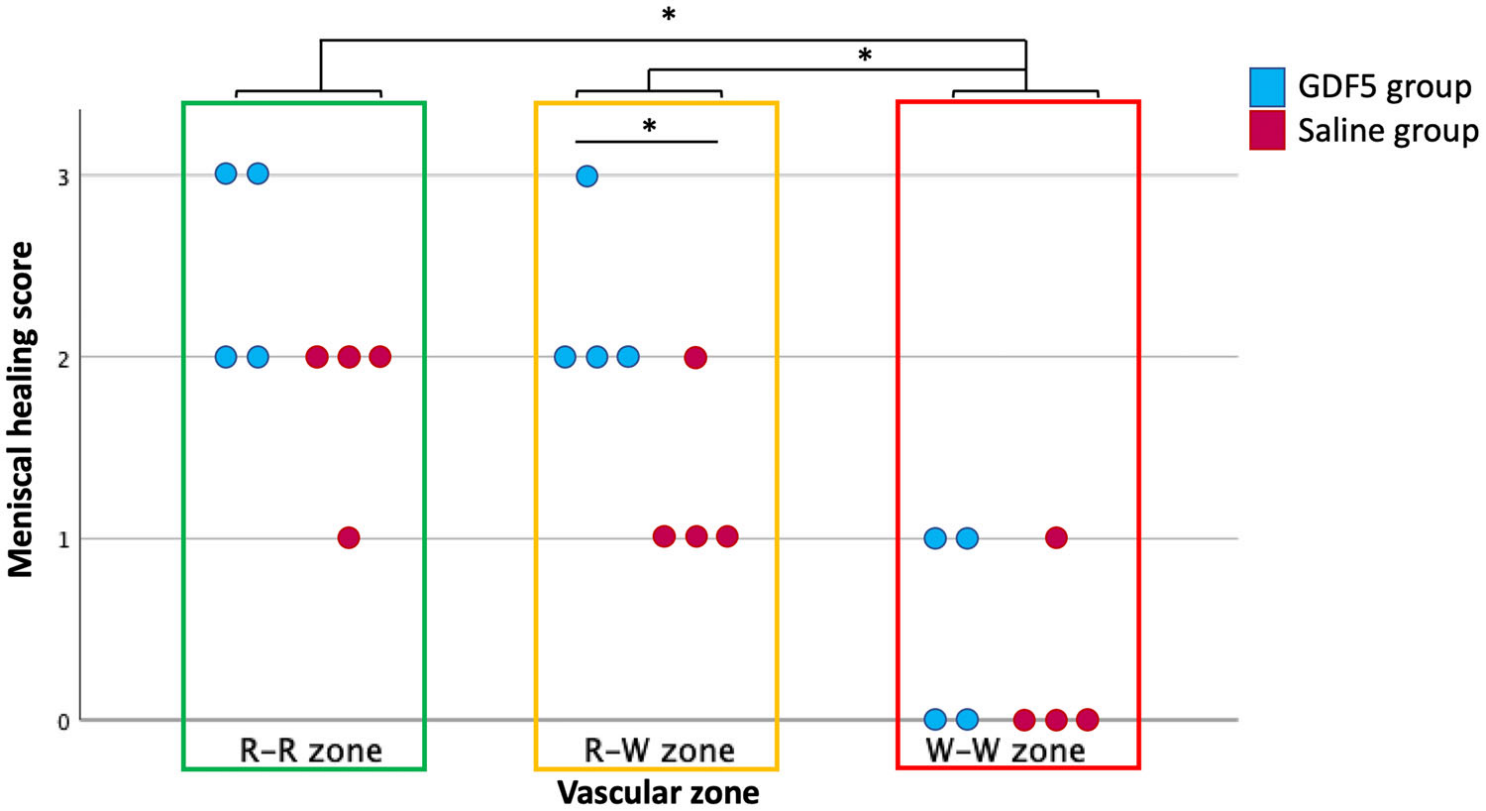
Meniscal healing score for each specimen in GDF5 and saline groups according to vascular zones. *p < 0.05. GDF5, growth differentiation factor 5; R‐R, red‐red; R‐W, red‐white; W‐W, white‐white.

The histological findings are shown in Figure 4. In the R‐R zone, two of the four menisci from the GDF5 group showed an MHS of 3, while no meniscus from the saline group reached this score. In the R‐W zone, the MHS (median [IQR]) in the GDF5 group (2 [2–2.75]) was statistically higher than in the saline group (1 [1–1.75]) (Figure 5). In the W‐W zone, only scores of 0 or 1 were observed. Detailed results are shown in Table 1. In the control group, without surgery, there were no meniscal tears so the MHS is not applicable. This control group ensures that no meniscal damage has occurred without surgery.

**Table 1:** Median [interquartile range] meniscus healing score for each group according to vascular zones.

Zones	GDF5 group	Saline group	*p* values	Effect size (*r*)
Red‐red	2.5 [2–3]	2 [1.25–2]	0.200	0.45
Red‐white	2 [2–2.75]	1 [1–1.75]	**0.047**	0.70
White‐white	0.5 [0–1]	0 [0–0.75]	0.686	0.14

*Note*: Effect sizes (*r*) are reported to quantify the magnitude of differences between groups. Bold is used to indicate a statistically significant result.

Abbreviation: GDF5, growth differentiation factor 5.

Regardless of the group, median [IQR] MHS gradually decreases from the R‐R zone (2 [2–2.75]) to the W‐W zone (0 [0–1]) (*p* < 0.001), as well as from the R‐W zone (2 [1–2]) to the W‐W zone (*p* < 0.001) (Figure 5).

The inter‐observer (0.88) and intra‐observer (0.90) ICC scores for meniscus healing were excellent and comparable to those of the study by Hashimoto et al. [14].

### Animal consideration

None of the rats used in the experiment had wound infections. One cutaneous wound dehiscence occurred on Day 1 and was treated immediately without any other complications. This animal was not excluded from the study, as wound dehiscence was only superficial. There were no major side effects following the use of GDF5 and no premature deaths were reported.

## DISCUSSION

The most significant finding of this study was that GDF5 enhances meniscal healing, particularly in the R‐W zone. The strongest effect was observed in this zone (*p* = 0.047, *r* = 0.70), suggesting that GDF5 significantly promotes healing in partially vascularized areas. In the R‐R zone, both groups showed high MHS, with a trend towards higher scores in the GDF5 group, although this was not statistically significant. Unfortunately, results were rather poor in the W‐W zone for both groups. Since the W‐W zone is also less cellularized, GDF5 cannot act sufficiently on the cells to perform its dedifferentiation role and thus produce extracellular matrix [6]. This supports that GDF5 enhances meniscal repair as long as vascularization and cells are present. This may explain why the W‐W zone remains a challenge.

Regardless of group assignment, there was a decrease in MHS as the distance from the periphery increased, similarly to humans [13, 17]. Tears in the R‐R zone mostly heal well, while those in the white‐white zone heal poorly, and those in the R‐W zone appear to have the potential for enhancement [1, 13, 17]. Better healing in the R‐W zone could mean a greater area that regains function after treatment. It remains to be determined whether the scar tissue is biomechanically effective.

GDF5 is a protein belonging to the bone morphogenetic protein family and the TGF‐beta superfamily with a significant role in bone and cartilage development and repair [27]. Loss of GDF5 function leads to severe syndromic skeletal malformations, such as Hunter‐Thompson or Grebe chondrodysplasia [39]. Some of the alleles that encode GDF5 are also associated with an increased risk of OA in the knee and hand [41]. Parish et al. reported on the efficacy of intra‐articular administration of recombinant human GDF5, to prevent and even reverse OA progression in a rat model [29]. GDF5 has also shown promising results in rat Achilles tendon healing, although its chondro‐inductive activity may not be entirely suited for tendon tissue [31]. The application of this factor has been extended to ligament healing, notably in rat knee collateral ligaments [38]. The addition of GDF5 has been shown to facilitate the healing process, both biomechanically and histologically, while a deficiency can delay early fracture healing. Although it does not compromise long‐term fracture healing, one study reported a delay in cartilage formation and remodelling [8]. Moreover, the expression of type I pro‐collagen genes is greater in tissues treated with GDF5 [38]. The role of GDF5 in the musculoskeletal system is well‐established; it can stimulate Type I collagen production by fibrochondrocytes, making it an ideal candidate for meniscal repair since the dry weight of the meniscus is predominantly composed of Type 1 collagen [3]. This was the primary motivation to use GDF5 for this study, although there were no applications described for the meniscus. This study suggests that GDF5 could be a promising factor for meniscus healing and warrants further attention, although current research is limited to animal and laboratory studies without human application [10, 31, 38]. Other growth factors, such as Vascular Endothelial Growth Factor and angiogenin, have been studied for meniscal healing, primarily aiming to enhance angiogenesis as a means to promote tissue repair [21, 22]. Fibroblast growth factor and connective tissue growth factor have also been investigated, targeting cells more directly, similar to our approach in this study [16, 28]. Among these diverse growth factor‐based strategies, none have demonstrated a truly significant effect on meniscal healing [26]. We hypothesize that by directly stimulating cellular activity and extracellular matrix production, GDF5 may offer a more targeted and efficient mechanism for promoting meniscal repair. A comprehensive understanding of all the factors involved in meniscal healing remains limited [40].

Enhancing meniscal repair remains a challenge, with numerous attempts at biological augmentation failing to identify a major factor of interest [26]. The findings of this study open the door to new research avenues, particularly if combining GDF5 with a scaffold, such as a hydrogel/adhesive matrix, to enable a more gradual and prolonged release of the factor [23]. The characteristics of the ideal adhesive for meniscal repair are well‐defined, and combining the adhesive with a factor that enhances meniscal healing could represent an innovative solution for meniscal repair [25]. No adverse effects have been associated with the use of GDF5, and its intra‐articular application has been shown to be safe. However, its effect on human cells needs to be tested before it can be considered for future clinical applications.

This study has some limitations, beginning with the small number of specimens allowed by a pilot study, but it represents the first step to justify a larger‐scale study. Additionally, the small size of rat menisci makes associated meniscal repair impossible, warranting a future larger animal model study. Another limitation is the greater potential for intrinsic healing of rats compared to humans, which may influence the translational relevance of our findings [7]. No biomechanical testing was performed due to the small size of the rat meniscus. Nonetheless, a small animal model study was necessary as proof of concept. Only a single post‐surgical injection was administered, despite human meniscal tears not being identified right after the trauma. With just one intra‐articular injection, the clearance of GDF5 may result in a transient effect. In addition, immediate post‐operative injection is prone to leakage outside the joint, even with hermetic closure. Future studies should investigate the effects of delayed injection, as well as evaluate the impact of varying GDF5 concentrations. The absence of a sham‐operated group is also a limitation which could have provided additional insight into potential effects related to the surgical incision itself. The vascular zones were chosen arbitrarily according to cutting depth, and the specifics of meniscal micro‐vascularization in rats are not known. Furthermore, only one time point was chosen before sacrifice, whereas multiple time points could be included in future studies.

## CONCLUSION

GDF5 can promote meniscus healing, especially in the R‐W zone, without adverse effect in a rat model. The W‐W avascular zone remains a challenge. The small sample size and absence of biomechanical evaluation are limitations that warrant caution when interpreting these findings. Further studies with larger sample sizes in bigger animal models, combined with meniscal repair, are required to confirm these preliminary results.

## AUTHOR CONTRIBUTIONS

David Mazy drafted the manuscript. David Mazy, Florina Moldovan and Marie‐Lyne Nault were responsible for research design. David Mazy Line Séguy, Nathaniel Léveillé and Irene Londono were responsible for data acquisition. David Mazy, Florina Moldovan and Marie‐Lyne Nault analyzed and interpreted the data. All authors reviewed and approved the final manuscript.

## CONFLICT OF INTEREST STATEMENT

Marie‐Lyne Nault: The institution (Hopital Sacré Coeur de Montréal) has received departmental funding for research and educational purposes from: Arthrex, Conmed, Depuy, Linvatec, Smith & Nephew, Stryker, Synthes, Tornier, Wright and Zimmer Biomet. Departmental funding was also provided to CHU Sainte‐Justine from Orthopaediatrics. The remaining authors declare no conflicts of interest.

## ETHICS STATEMENT

This study was approved by our institution's Animal Care and Experimentation Committee (project #2024‐5181, June 15, 2023).

## Data Availability

The data sets used and/or analyzed during the current study are available from the corresponding author on reasonable request.
